# Supplementary material for: HIVprotI: an integrated web based platform for prediction and design of HIV proteins inhibitors
Source: J Cheminform. 2018 Mar 9;10:12. doi: 10.1186/s13321-018-0266-y (PMC5845081; doi:10.1186/s13321-018-0266-y)
Supplement: Supplementary file 2 — Additional file 2. Source code of HIVProtI web server. [file 13321_2018_266_MOESM2_ESM.zip › HIVprotI_Source-code/hivprotI/weka-3-6-6/doc/weka/core/TechnicalInformationHandler.html]

TechnicalInformationHandler


---


|  |  |  |
| --- | --- | --- |
| **Overview** \| **Package** \| **Class** \| **Tree** \| **Deprecated** \| **Index** \| **Help** \| **Weka's home** | |  |
| **PREV CLASS**   **NEXT CLASS** | **FRAMES**    **NO FRAMES**     **All Classes** |  |
| SUMMARY: NESTED \| FIELD \| CONSTR \| METHOD | DETAIL: FIELD \| CONSTR \| METHOD |  |


---


## weka.core Interface TechnicalInformationHandler

**All Known Implementing Classes:**: AdaBoostM1, AdditiveRegression, ADNode, ADTree, Agrawal, AODE, AODEsr, Apriori, Bagging, BallTree, BayesianLogisticRegression, BFTree, BIFReader, BIRCHCluster, BottomUpConstructor, BoundaryVisualizer, BVDecompose, BVDecomposeSegCVSub, CfsSubsetEval, ChebyshevDistance, ChisqMixture, CitationKNN, ClassBalancedND, ClassificationViaRegression, CLOPE, Cobweb, ComplementNaiveBayes, ConsistencySubsetEval, CoverTree, CVParameterSelection, Dagging, DataNearBalancedND, DBScan, DecisionTable, Decorate, Discretize, DMNBtext, DTNB, END, EuclideanDistance, FarthestFirst, FPGrowth, FT, GaussianProcesses, GeneralizedSequentialPatterns, GeneticSearch, Grading, HNB, IB1, IBk, Id3, IteratedLovinsStemmer, J48, J48graft, JRip, K2, K2, KDTree, KernelFilter, KMeansInpiredMethod, KStar, LADTree, LBR, LeastMedSq, LED24, LibLINEAR, LibSVM, LinearForwardSelection, LMT, Logistic, LogitBoost, LovinsStemmer, LWL, M5Base, M5P, M5Rules, ManhattanDistance, MDD, MedianDistanceFromArbitraryPoint, MedianOfWidestDimension, MedianOfWidestDimension, MetaCost, MIBoost, MIDD, MiddleOutConstructor, MidPointOfWidestDimension, MIEMDD, MINND, MIOptimalBall, MISMO, MISVM, MIWrapper, MixtureDistribution, MultiBoostAB, NaiveBayes, NaiveBayesMultinomial, NaiveBayesMultinomialUpdateable, NaiveBayesSimple, NaiveBayesUpdateable, NBTree, ND, NNge, NominalToBinary, NormalMixture, OneR, OPTICS, Optimization, OrdinalClassClassifier, PaceRegression, PairedCorrectedTTester, PART, PKIDiscretize, PLSFilter, PointsClosestToFurthestChildren, PredictiveApriori, Prism, Puk, RacedIncrementalLogitBoost, RaceSearch, RandomForest, RandomProjection, RandomSearch, RandomSubSpace, RankSearch, RegSMO, RegSMOImproved, RELAGGS, ReliefFAttributeEval, RotationForest, ScatterSearchV1, sIB, SimpleCart, SimpleLogistic, SimulatedAnnealing, SimulatedAnnealing, SlidingMidPointOfWidestSide, SMO, SMOreg, SMOTE, SPegasos, Stacking, StackingC, StringKernel, SVMAttributeEval, TabuSearch, TabuSearch, TAN, TAN, Tertius, TopDownConstructor, UserClassifier, VFI, Vote, VotedPerceptron, WAODE, Wavelet, Winnow, WrapperSubsetEval, XMeans

---

``` public interface TechnicalInformationHandler ```

For classes that are based on some kind of publications. They return
a TechnicalInformation object filled with the data of the publication.

**Version:**
:   $Revision: 1.1 $

**Author:**
:   fracpete (fracpete at waikato dot ac dot nz)

**See Also:**: `TechnicalInformation`

---

| **Method Summary** | |
| --- | --- |
| `TechnicalInformation` | `getTechnicalInformation()`             Returns an instance of a TechnicalInformation object, containing detailed information about the technical background of this class, e.g., paper reference or book this class is based on. |

**Method Detail**

### getTechnicalInformation

```
TechnicalInformation getTechnicalInformation()
```

:   Returns an instance of a TechnicalInformation object, containing
    detailed information about the technical background of this class,
    e.g., paper reference or book this class is based on.

    :   **Returns:**: the technical information about this class


---


|  |  |  |
| --- | --- | --- |
| **Overview** \| **Package** \| **Class** \| **Tree** \| **Deprecated** \| **Index** \| **Help** \| **Weka's home** | |  |
| **PREV CLASS**   **NEXT CLASS** | **FRAMES**    **NO FRAMES**     **All Classes** |  |
| SUMMARY: NESTED \| FIELD \| CONSTR \| METHOD | DETAIL: FIELD \| CONSTR \| METHOD |  |


---
